# The Progress of Cancer Research

**Published:** 1908-11-07

**Authors:** 


### The Hospital

A Journal of

The Medical Sciences and Hospital Administration.

New Series. No. 87, Vol. IV. [No. 1159, Vol. XLV.] SATURDAY, NOVEMBER 7, 1908.

### The Progress of Cancer Research.

The Imperial Cancer Research Fund has issued its Third Scientific Report, after a silence of three years' duration. The first was issued in 1904, the second a year later. Annual issues were then discarded, and the whole attention of the investigators of the fund directed towards the accumulation of new facts. Those who read the bulky volume of some five hundred pages which constitutes this third report will approve the self-denying ordinance which has enjoined so prolonged a silence upon the official workers in this great field. The lapse of time has enabled them to extend the evidence available in 1905 by observations upon no fewer than seventy propagable malignant new growths of the mouse. These include not only carcinomata and malignant adenomata, but sarcomata of all kinds.

In the Second Scientific Report the more likely theories advanced in explanation of cancer were reviewed and discarded in the light of experiment. It is in a way satisfactory, as instancing capacity to weigh evidence, that the investigators of the fund have not had occasion to revise their judgment upon these hypotheses. Dr. Bashford in his introduction to the current report devotes some pains to the further demolition of one or two theories which appear still to be exercising a baneful influence upon the outlook of cancer research. In particular he cites the theory vaguely held by a good many people that cancer is "congenital," and produced by the disordered development of embryonic cells. "The study of the incidence of cancer as determined by irritants in man," he says, "demonstrates absolutely that the generalisation of the idea of a congenital or embryonic origin is incorrect." This comforting reflection is borne out by two novel items of evidence from India. Cancer of the skin of the abdomen, which is almost unknown in Europe, is very common in Kashmir. It is, of course, incredible that this should indicate a peculiar distribution of the hypothetical embryonic tissue among the natives of Kashmir; on the other hand there is a perfectly acceptable explanation to be found in the local custom of carrying at the waist a small earthenware vessel enclosed in basket-work, and containing a charcoal fire. This little charcoal oven is called a Kangri, and the effect of the repeated burns and chronic irritation resulting from the custom of thus carrying it is the "Kangri ulcer," which passes on occasion into the "Kangri cancer." The latter is a typical squamous-celled carcinoma, as may be seen from an excellent photograph supplied with the text. The second of the newer arguments against the "congenital" theory is the frequency with which the women of India suffer from cancer of the inside of the mouth, while in Europe cancer among females rarely manifests itself in this situation. The explanation lies in the custom common to Indian women of chewing a mixture of betel-nut, areca-nut, slaked lime, and tobacco, and of sleeping with the plug at precisely that spot in the cheek at which cancer commonly appears. As Dr. Bashford says, if the putative congenital germs of cancer are to be postulated to explain facts like these, then we must also assume them to be distributed uniformly about the body, and this makes the so-called explanation a very shadowy one indeed.

The possible influence of heredity in determining the appearance of cancer has long formed a subject for speculation, but the difficulty of carrying out investigations in the case of a long-lived species like man has precluded anything like definite information. It appears, however, that the subject is now in a fair way towards being settled by means of accurate experiments upon mice. The surgical removal of spontaneously-occurring mammary cancers in mice and subsequent breeding from the affected animals has supplied over a thousand mice of known cancerous parentage. By successively crossing other spontaneously-affected animals with the offspring of cancerous parents the cancerous heredity is intensified to a known extent. Up to the present time there is no evidence that the in-breeding of cancerous mice has enhanced the liability of the offspring to mammary carcinoma.

We have drawn attention to one or two of the points in the report likely to be of general interest, but to the specialist the whole of it will appeal. It is true that at present no far-reaching generalisation has been evolved to link together the rapidly-accumulating mass of facts; but this, so far from causing disappointment, may well form matter for congratulation; for we have suffered much at the hands of false prophets in the matter of cancer, and the wise reticence of Dr. Bashford and his colleagues will, we have no doubt, simply serve to increase the general confidence in them. Although we are told that no practical outcome to the experimental investigation is yet in sight, there is one item of information which is certain to attract much attention. It is that the inoculation of normal skin will protect practically every mouse against a subsequent inoculation of squamous-celled carcinoma. Nevertheless, once inoculation has been successful and a carcinoma of the skin has resulted, subsequent inoculations of normal skin cease to be protective against secondary inoculations of the cancer. It appears, therefore, that the presence of an inoculated tumour produces some constitutional modification favourable to dissemination, and that in the circumstances determining this modification may lie some clue to the prevention of secondary growths in man. But the General Superintendent enters his *caveat* against premature application to the treatment of human cancer of any of the results of animal experiment save one. This is that the rationale of the early surgical removal of primary growths is amply justified, and that no substitute for it has yet been discovered.

